# Dissecting the Role of Circular RNAs in Sarcomas with Emphasis on Osteosarcomas

**DOI:** 10.3390/biomedicines9111642

**Published:** 2021-11-08

**Authors:** Eleftheria Lakiotaki, Dimitrios S. Kanakoglou, Andromachi Pampalou, Eleni A. Karatrasoglou, Christina Piperi, Penelope Korkolopoulou

**Affiliations:** 1 First Department of Pathology, Medical School, National and Kapodistrian University of Athens, 75 Mikras Asias Street, 11527 Athens, Greece; kanakoglou.d@gmail.com (D.S.K.); apampalou@gmail.com (A.P.); elina_karat@hotmail.com (E.A.K.); pkorkol@med.uoa.gr (P.K.); 2 Department of Biological Chemistry, Medical School, National and Kapodistrian University of Athens, 75 Mikras Asias Street, 11527 Athens, Greece; cpiperi@med.uoa.gr

**Keywords:** circular RNAs, osteosarcoma, Kaposi sarcoma, rhabdomyosarcoma, gastrointestinal stromal tumors, diagnosis, therapy

## Abstract

Circular RNAs (circRNAs) are single-stranded RNAs generated from exons back-splicing from a single pre-mRNA, forming covalently closed loop structures which lack 5′-3′-polarity or polyadenylated tail. Ongoing research depicts that circRNAs play a pivotal role in tumorigenesis, tumor progression, metastatic potential and chemoresistance by regulating transcription, microRNA (miRNA) sponging, RNA-binding protein interactions, alternative splicing and to a lesser degree, protein coding. Sarcomas are rare malignant tumors stemming from mesenchymal cells. Due to their clinically insidious onset, they often present at advanced stage and their treatment may require aggressive chemotherapeutic or surgical options. This review is mainly focused on the regulatory functions of circRNAs on osteosarcoma progression and their potential role as biomarkers, an area which has prompted lately extensive research. The attributed oncogenic role of circRNAs on other mesenchymal tumors such as Kaposi Sarcoma (KS), Rhabdomyosarcoma (RMS) or Gastrointestinal Stromal Tumors (GISTs) is also described. The involvement of circRNAs on sarcoma oncogenesis and relevant emerging diagnostic, prognostic and therapeutic applications are expected to gain more research interest in the future.

## 1. Introduction

Sarcomas are rare tumors, with an incidence between 1 to 5 per 1,000,000 population, accounting for over 20% of all pediatric solid malignant tumors and less than 1% of all adult solid malignant tumors [1,2]. The most common types are reportedly leiomyosarcomas, Kaposi Sarcomas (KS), Undifferentiated Pleomorphic Sarcomas (UPS), liposarcomas and fibrosarcomas [2]. Osteosarcoma (OS) is the most frequent malignant bone tumor, occurring in all age groups, with the highest incidence been detected at 5–29 years of age and a second peak occurring above 50 years, following a bimodal distribution [2,3]. It usually affects the metaphysis of the limbs, such as the distal femur, humerus and proximal tibia [3]. Its treatment includes surgery and chemotherapy (adjuvant and neoadjuvant). Cure rates have improved from 20% to 70% for patients without metastasis [4,5]; however, patients with metastatic OS or patients who have developed chemoresistance experience poor survival rates [6].

KS is a very common tumor, of intermediate malignant potential, in patients with AIDS in the United States, and also common in patients with underlying immunodeficiency, such as organ transplant recipients [7,8], representing one of the most frequent tumors overall [2]. It is a vascular tumor driven by the Kaposi’s Sarcoma-Associated Herpesvirus (KSHV/HHV-8) which arises mainly in the skin, lymph nodes and mucous membranes, while it can also affect the majority of the visceral organs [9,10].

Rhabdomyosarcoma (RMS) comprises 5% of all pediatric tumors [11], and stems from mesenchymal cells with skeletal muscle differentiation [12]. It comprises several histological subtypes. Embryonal RMS (ERMS) is the most common soft tissue sarcoma in children and adolescents (4.5 cases/million people aged <20 years) [13]. Alveolar RMS (ARMS) is the second most common histologic subtype, occurring in a slightly older population in comparison to ERMS [14]. Pleomorphic RMS (PRMS) usually presents in adults [15], whereas Spindle cell/sclerosing RMS is the rarest histologic subtype and can affect all age groups [16]. Gastrointestinal Stromal Tumors (GISTs) can be found throughout the gastrointestinal tract, with the stomach being the most common site (54% of all GISTs), but also in extragastrointestinal locations, mainly in the mesentery, omentum and retroperitoneum. The incidence is about 10–15 cases per million per year [17]. This category of tumors is characterized by gain-of-function mutations of the *KIT* (KIT Proto-Oncogene, Receptor Tyrosine Kinase) or *PDGFRA* (Platelet Derived Growth Factor Receptor Alpha) oncogene. However, GISTs that are wild-type for *KIT* or *PDGFRA* are characterized by alterations in Succinate Dehydrogenase (SDH) subunit genes [18,19,20]. The assessment of prognosis is estimated by taking into account several parameters, namely tumor size (using as cutoff values 2 cm, 5 cm and 10 cm), mitotic count (lower or higher than 5 mitoses/5 mm^2^), and location (gastric vs. non-gastric) [21]. Complete surgical resection is the primary treatment for localized tumors [22], while imatinib, the tyrosine kinase inhibitor of *KIT* and *PDGFRA* receptors is considered the standard treatment for metastatic or unresectable GISTs [23,24].

Circular RNAs (circRNAs) represent a category of non-coding RNAs (ncRNAs) and constitute functional RNAs that are predetermined not to be translated, but transcribed. CircRNAs are characterized by single-stranded closed-loop structures without 5′-3′-polarity or a polyadenylated tail [25,26,27,28]. The term “circRNA” was first used by Sanger et al. when identifying the structure of viroids [29]. Their annular structure makes them more stable and resistant to degradation by exonucleases such as Ribonuclease (RNase) R, exhibiting longer half-life than linear RNA [25,27]. Upon their discovery, circRNAs were thought to be the byproducts of splicing errors and not given appropriate attention. After the advances in high-throughput RNA-sequencing technologies and bioinformatics, it has been shown that the expression of circRNAs accounts for more than 10% of gene expression [26,30,31]. Taking into account that their expression is tissue, cell or developmental stage-specific and also regulated by certain conditions such as stress, compelling evidence points towards circRNAs playing an important role in human tumorigenicity, tumor progression, metastatic spread and signaling pathway modulation [32,33,34,35].

Although the bulk of the research regarding circRNAs has been focused on carcinomas, there are reports illustrating the role of circRNAs on sarcomagenesis, with the majority of experimental work concentrating on OS. In this review, we provide an update on the current knowledge related to circRNAs’ role on sarcoma tumorigenesis, describing their implication in various tumor types.

## 2. Main Characteristics of CircRNAs

### 2.1. Categories, Biogenesis, Localization, Degradation

CircRNAs are categorized according to their synthesis by introns, exons, or both into four main categories described below:

1. Exonic circRNAs (EcircRNAs), localized to the cytoplasm [26] and consisting of a single or multiple exons, usually two or three [31,36]. A median length of 353 nucleotides is required for single-exon back-splicing.
2. Intronic circRNAs (ciRNAs), localized to the cell nucleus [37].
3. Exon-Intron circRNAs (EIcircRNAs), also localized to the cell nucleus and functionally similar to ciRNAs [38].
4. Intergenic circRNAs, generated by two ciRNAs fragments flanked by GT-AC splicing signals, acting as the splice donor and acceptor of the circular junction, and forming an integrated circRNAs [39].

**Figure 1:**
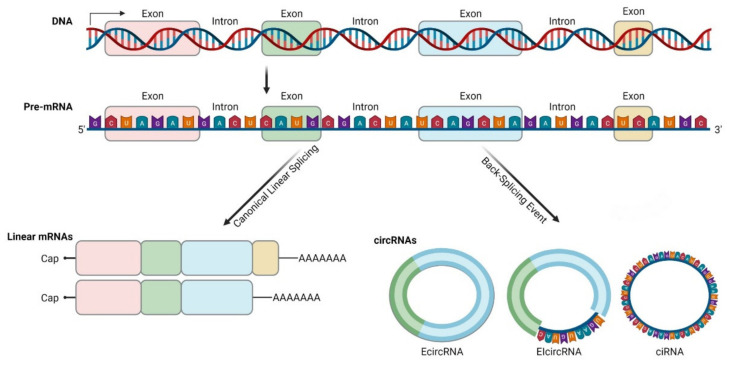
Biogenesis and diversity of circRNAs. CircRNAs are produced by exon-skipping or another non-canonical event (like back-splicing) that can initiate internal splicing. CircRNAs are present in blood, body fluids and tissues, and can serve as potential disease biomarkers. They are non-polyadenylated, unlike mRNAs, and they are covalently closed. CircRNAs are resistant to exonucleases digestion due to their lack of 5′ or 3′ end. CircRNAs can be exonic, intronic or a combination of both (this illustration was created with BioRender.com, accessed on 3 November 2021).

CircRNAs are generated by back-splicing (Figure 1) of primary messenger RNA (mRNA) transcripts. During back-splicing, a 5′ splice site (donor) is joined to an upstream 3′ site (acceptor) by the spliceosome machinery, forming a covalently closed structure with a specific junction site [37]. This process competes with canonical splicing and can produce different circRNAs from the same sequence [30,40]. It has been reported that the more back-splicing an exon can undergo, the less it is included in the fully processed mRNA [41].

Back-splicing occurs both co-transcriptionally and post-transcriptionally and is a much less efficient process than canonical splicing. It is favored over canonical splicing up on depletion of splicing factors and can be affected by epigenetic changes within histones and gene bodies [30,42,43,44].

There are three basic models of circRNAs biogenesis. Firstly, on an exon-skipping event, lariats are formed when alternative exons are spliced out of the final mRNA product. The lariat containing the excised exons undergoes internal back-splicing and circRNA (EcircRNA or EIcircRNA) are formed [41,45]. Intron lariats that contain a ciRNA specific consensus motif consisting of an 11-Nucleotide (nt) C-rich element near the branch point and a 7-nt GU-rich element near the 5′ splice site can escape debranching and form ciRNAs according to this model [46]. Secondly, on “intron pairing-driven circularization”, inverted repeat elements (for example ALU elements) are located in the upstream and downstream introns. By base-pairing of complementary sequences of inverted repeat elements, a hairpin structure is formed and looping brings the acceptor and donor sites in proximity [36,37,41]. Thirdly, looping can be mediated by RNA-Binding Proteins (RBPs), such as Quaking, Muscleblind or FUS/TLS (Fused in Sarcoma/Translocated in Liposarcoma) protein [47,48,49]. Interestingly, not all RBPs promote circRNAs biogenesis, as ADAR1 (Adenosine Deaminase RNA-Specific Binding Protein) binds to double-stranded RNA regions and promotes the melting of stem structures to destabilize RNA pairing, thereby suppressing circRNAs formation [50].

Apart from the four main categories, there are newly emerging subtypes of circRNAs, such as tRNA Intronic circRNAs (tricRNAs), generated during pre-tRNA maturation or fusion circRNAs (f-circRNAs), created from chromosomal translocations in host genes [51,52].

Following synthesis, all circRNAs, apart from intron-containing, are transported from the nucleus to the cytoplasm in a size-dependent manner by the enzymes Adenosine Triphosphate (ATP)-dependent RNA helicase DDX39A and spliceosome RNA helicase DDX39B [53].

First reports regarding the mechanisms implicated on circRNAs degradation were based on studies of the circRNAs Cerebellar Degeneration-Related Protein 1 Transcript (CDR1as), a circRNA upregulated in OS, that described cleavage of CDR1as as mediated by Argonaute 2 (Ago2), after binding to microRNA (miRNA) miR-671 [54]. Nevertheless, whether this type of degradation is universal to circRNAs is still not clear [30]. Recent evidence points to other endonucleases functioning on circRNAs decay, upon special conditions. A portion of circRNAs that have undergone m^6^A (N6-Methyladenosine) modification are cleaved by the ribonuclease complex RNase P/MRP, a process mediated by the m^6^A reader protein YTHDF2 (YTH N6-Methyladenosine RNA Binding Protein 2) and HRSP12 (Heat-Responsive Protein 12) [55]. That could be the case for circNRIP1, a circRNA upregulated in OS. Its expression was reportedly elevated by METTL3, a methyltransferase that induces m^6^A modification to circNRIP1. In turn, circNRIP1 sponges miR-199a to upregulate FOXC2 expression in OS [56]. Additionally, RNase L, activated upon viral infection, can degrade all types of circRNAs. UPF1 (ATP-Dependent RNA Helicase Upstream Frameshift 1) and associated endonuclease G3BP1 (Ras Gtpase-Activating Protein-Binding Protein 1) can target and catalyze the degradation of some circRNAs [57]. Moreover, circRNAs accumulation is eliminated through exocytosis or exosome activity [58].

### 2.2. Biochemical Properties and Detection Methods

CircRNAs possess an exceptionally stable structure that results in their accumulation to the cytoplasm as previously mentioned, along with possible mechanisms of degradation. Most EcircRNAs’ half-life surpasses 48 h, in contrast to linear mRNA with average half-life of 10 h [26].

Another feature of circRNAs is their tissue -and developmental stage- or age-specificity. In human tissues, RNA-sequencing studies demonstrated that up to 50% of circRNAs were expressed in a highly specific pattern and showed increased levels on fetal compared to adult tissues [59].

CircRNAs are abundant in human tissues and tend to accumulate in tissues with low proliferation rate, for example cardiomyocytes. CircRNAs accumulation is also dependent on age [60,61]. Different human tissues show variable rates of circRNAs production, as in the human heart where 9% of the expressed genes produce circRNAs, whereas in the human brain the rate of production reaches 20% [62]. However, circRNA expression is not related to that of its linear isoforms, and under specific circumstances can far exceed it [30].

CircRNAs’ stability to degradation and the lack of polar structure at the end are properties that can be utilized for their detection. Their migration rate in a cross-linked gel is slower than that of long linear RNAs, and when compared with homologous gene transcription, nucleic acids show slower migration rate in weakly cross-linked gels, enabling circRNAs detection with northern blot analysis [25,63]. Subcellular location can be assessed with the Fluorescence In Situ Hybridization (FISH) technique [38,46]. Improved algorithms for high-throughput sequencing (circRNA candidate sequence boundary combination concerning different forms of exon rearrangement in comparison to sequencing data, different sequence alignment algorithms to match sequencing data to the genomic sequence and direct detection from cDNA sequences by designing multiple splice sequences) have also rendered the detection of low-abundance circRNAs feasible [64,65]. Currently, multiple circRNA-associated in silico tools and pipelines can support the de novo identification, assembly and annotation of circRNAs [66]. Depending on their implementation, circRNA identification tools are divided into three categories; BSJ-based (Back-Splicing Junction), integrated-based and machine learning-based [67]. Additionally, novel tools can perform more complex functions (e.g., alternative splicing events, expression estimation and circRNA structure prediction). In their review, Chen et al. [67] provide a comprehensive guide for hundreds of circRNA-related tools and their functionality.

### 2.3. Biological Functions

**Figure 2:**
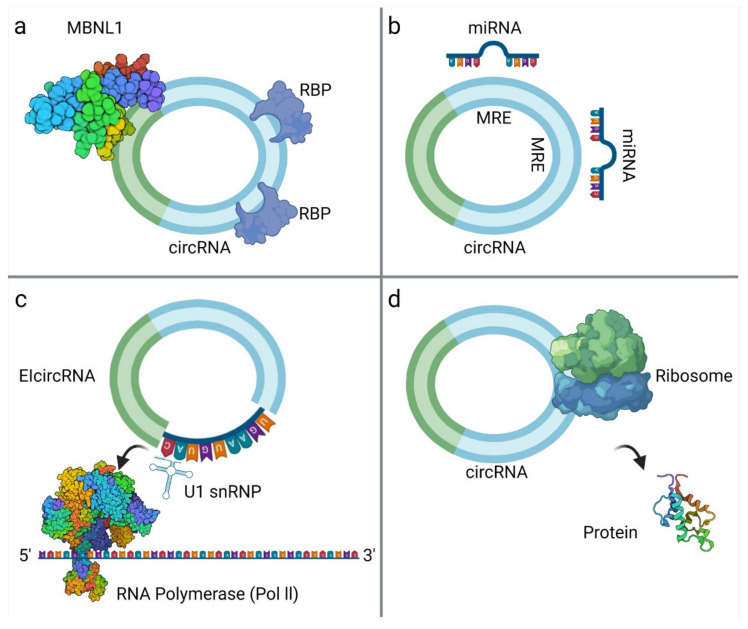
CircRNA interactions: (**a**) CircRNA binds with RNA-Binding Proteins (RBPs), such as MBNL1 [80]; (**b**) circRNAs act as miRNA sponges; (**c**) EIcircRNA interacts with the U1 Small Nuclear Ribonucleoprotein (snRNP) and then binds to RNA Polymerase II (Pol II) [81]; (**d**) circRNA translation into proteins. The surface of the molecules MBNL1 and Pol II are modelled according to van der Waals surface (this illustration was created with BioRender.com, accessed on 3 November 2021).

CircRNAs exert many important biological functions through three major mechanisms of action (Figure 2):

1. MiRNA sponging
2. Protein and mRNA interplay
3. Translation

MiRNA sponging is the most well-studied mechanism of action of circRNAs. CircRNAs contain binding sites for miRNAs and compete endogenous RNAs to sequester miRNAs via complementary base-pairing, while inhibiting miRNA from binding to their target molecules [68]. Subsequently, miRNA sponging leads to suppression of target mRNAs. The well-known paradigm of circRNA CDR1as, with more than 70 binding sites for miR-7 [69], but also other circRNAs such as circRNA-Homeodomain-Interacting Protein Kinase-2 (circHIPK2), have mechanistically confirmed sponge effect to miR124-2HG [70]. Considering their multiple binding sites, circRNAs can act both as oncogenes or tumor suppressors through binding to different miRNAs [37]. A large part of research focusing on circRNA role on sarcomas refers to circRNA-miRNA interactions and their impact on tumor growth, described later. Nevertheless, bioinformatics analysis reports that although circRNAs may have abundant binding sites, they may not exert prominent sponge effect, and under normal conditions this effect is not important on highly expressed miRNAs [31,54,71,72].

CircRNAs interactions with proteins are multifold, since they can act as protein sponges, scaffolds, enhance protein function or recruit proteins to specific subcellular compartments, or even facilitate contact between two or more proteins. It is well documented that circRNAs that carry RBP binding motifs may act as decoys or sponges to RBPs and interfere with their effects [37]. CircMbl is documented to bind both mbl and MBNL1 (Muscleblind-like Protein 1) proteins. Upon increased mbl or MBNL1 proteins, circMbl synthesis is promoted. In turn, linear mRNA splicing of the gene is favored, creating an autoregulatory loop of the gene expression [47]. In addition, RNA Polymerase II-U1 (Pol II-U1) Small Nuclear Ribonucleoproteins (snRNPs) complex binding is facilitated by interaction with circEIF3J with U1 RNA, resulting in upregulated parental gene transcription [38]. Similarly, circRNAs can bind dsRBPs [Double-Strand RNA-Binding Proteins (e.g., NF90, NF110)] [73] and create a reservoir to be used in specific conditions. Moreover, several circRNAs are reported to act as mRNA decoys or modulate mRNA stability [54,74]. Reports of proteins binding circRNAs without predicted binding sites imply that the tertiary structure of circRNAs may play an important role on protein binding, and that the tertiary structure may exhibit certain fluidity in different developmental stages or tissues [75]. The mechanisms underlying the way that tertiary structure affects circRNA function should be an object of further research.

Bioinformatics have proven that circRNAs have Open Reading Frame (ORF) and ribosome binding site, making translation possible. Translation may occur via Internal Ribosome Entry Site (IRES) elements that recruit the ribosomal 40S subunits in a cap-independent manner [76]. m^6^A modification may also play a role in circRNA translation, as m^6^A reader protein YTHDF3 interacts with translation initiation factors elF4G2 and elF3A, after binding to m^6^A modified circRNA [59]. The resulting peptides are truncated versions of the original proteins and, therefore, their functional relevance remains to be investigated. They might serve as dominant-negative protein variants, having been expressed under different conditions than the original protein or localized to other cellular compartments [76].

It has been mentioned above that circRNAs that localize in the nucleus (EIcircRNAs and ciRNAs) can regulate their parental gene expression by interacting with U1 snRNP complex. CircRNAs also regulate alternative splicing and transcription, given that under certain circumstances (depletion of splicing factors) circRNA biosynthesis is promoted over canonical splicing [47], exerting an important role on translation regulation by interfering with mRNA mobility and stability [54,74,77]. Other studies also show that circRNAs control the role of ribosomes in protein expression [78]. In addition, recent data demonstrate that stabilized circRNAs could form circRNA pseudogenes and be retrotranscribed and integrated into the genome [27,79], thus modifying gene expression.

### 2.4. Roles in Homeostasis and Malignancy

CircRNAs participate in cell cycle regulation as key factors in multiple check points [82] and modulate c-MYC expression as well as other molecules downstream of c-MYC, including p21. They have also been involved in mediation of immune response [83], cell differentiation and pluripotency [84], as well as apoptosis [85].

Recent literature points to the dysregulation of circRNAs in cancer, inducing oncogenic or suppressive effects and playing a pivotal role in tumorigenesis [82]. Bioinformatics analysis has yielded interesting results on many tumor types, identifying a network of circRNAs and their target miRNAs and mRNAs as well as their interaction with important signaling pathways, such as Wnt/β-catenin, TGF-β (Transforming Growth Factor Beta) or PI3K/AKT [82]. They have also been shown to regulate Epithelial-Mesenchymal Transition (EMT), resist apoptosis, induce angiogenesis, DNA methylation and demethylation coordination as well as influence metastatic spread [68,82], whereas some studies have indicated the presence of circRNAs in exosomes, implicating their transport between cells and tissues [30,34,86]. Moreover, new evidence implies that circRNAs may affect acquired chemoresistance [87]. The above-mentioned mechanisms have been shown to be critical in sarcomagenesis [88,89,90,91,92,93,94], and a large number of circRNAs have been demonstrated to affect chemoresistance in OS, such as hsa_circ_0004674, hsa_circ_0081001 and circPVT1, among others [88,95,96,97,98].

## 3. CircRNAs Functions in Osteosarcoma

### 3.1. Upregulated CircRNAs in OS

Currently there has been an ever-increasing number of studies depicting the role of circRNAs as tumor promoters in OS. CircRNAs, by binding to multiple miRNAs and other proteins, interfere with numerous signaling pathways and promote cell proliferation, invasion, migration, chemoresistance and apoptosis inhibition. A detailed description of the current literature on upregulated circRNAs in OS is given in Table 1.

### 3.2. Downregulated CircRNAs in OS

The number of downregulated circRNAs in OS is much lower in comparison to upregulated circRNAs; however, there are reports of circRNAs involved in chemotherapy resensitization and tumor growth inhibition. A detailed description of the current literature on downregulated circRNAs in OS is given in Table 2.

### 3.3. CircRNAs of Ambiguous Significance in OS

This section provides an account of certain circRNAs detected either upregulated or downregulated in different studies, with a consequent effect on OS tumor growth and prognostic parameters. The discrepancy observed among experimental findings might be attributed to sample numbers and types (fresh, frozen) as well as to intrinsic heterogeneity between cell lines and patient tissues or to differences in methodology employed.

#### 3.3.1. hsa_circ_0002052

The study of Wu et al. demonstrated that hsa_circ_0002052 is significantly downregulated in OS patient tissues and cell lines (143B, MG-63, HOS and U2OS). Overexpression of hsa_circ_0002052 inhibited cell proliferation, migration, and invasion in vitro and blocked cell growth in vivo [90]. hsa_circ_0002052 suppressed the activation of Wnt/β-catenin pathway by sponging miR-1205, and rescuing APC2 expression, which is a tumor suppressor often detected in many tumors [79,224,225] that regulates negatively the Wnt/β-catenin pathway [90]. Moreover, low levels of hsa_circ_0002052 were correlated with worse survival and worse PFS (Progression-Free Survival) [90].

In contrast, Zhang et al. reported that hsa_circ_0002052 is upregulated in OS tissues and is related to advanced stage, tumor size and metastasis, as well as to low survival rate. In addition, by sponging miR-382, hsa_circ_0002052 enhanced STX6 expression, resulting in Wnt/β-catenin pathway activation [214,226]. These utterly opposite results need further investigation to elucidate the role of hsa_circ_0002052 in OS.

#### 3.3.2. circ-ITCH

circ-ITCH was reported as a tumor suppressor by the study of Ren et al., who detected downregulation of circ-ITCH on OS tissues and cell lines (MG-63, U2OS, SaOS-2) [227]. circ-ITCH overexpression sponged miR-22 and promoted apoptosis, while blocking cell viability, proliferation, migration and invasion [227]. miR-22 inhibition suppressed PTEN/PI3K/AKT and SP-1 pathways in OS [227].

Li et al. investigated circ-ITCH expression in OS cell lines SJSA-1 and U2OS and detected upregulated levels. Functional studies demonstrated that circ-ITCH participates in circ-ITCH/miR-7/EGFR pathway to promote OS cell migration, invasion and growth. Specifically, circ-ITCH acted as decoy to miR-7 and supports high levels of EGFR, while its migratory-invasive capacity was dependent to EGFR phosphorylation [228].

Zhou et al. also detected downregulation of circ-ITCH in OS tissues and cell lines. They detected a correlation of low circ-ITCH levels with promotion of OS development in OS tissues and MG-63 and KHOS cell lines, as well as with contribution to DXR (Doxorubicin) chemoresistance via the miR-524/RASSF6 axis [229].

#### 3.3.3. circ_HIPK3

circ_HIPK3 was reported to be downregulated in OS tissues, plasma and OS cell lines (SaOS-2, HOS, KHOS, MG-63, 143B, U2OS). circ_HIPK3 levels were correlated with Enneking stage and lung metastasis [230]. Furthermore, low circ_HIPK3 levels correlated with shorter overall survival and poor prognosis. Enhancement of circ_HIPK3 blocked OS cell proliferation, migration and invasion in vitro [230].

On the other hand, Huang suggested that circ_HIPK3 can promote metastasis in OS by showing that circ_HIPK3 promotes migration, invasion and growth in OS tissues and cell lines (U2OS, SW1353) [231]. circ_HIPK3 could bind and inhibit miR-637 and further elevate STAT3 (Signal Transducer and Activator of Transcription 3) expression to exert oncogenic effects [231].

Wen et al. showed that circ_HIPK3 was upregulated in OS tissues and cell lines (HOS, MG-63, U2OS, SJSA-1 OS) and demonstrated that modulation of miR-637/HDAC4 axis promoted proliferation, migration and invasion in OS [232].

#### 3.3.4. hsa_circ_0102049

hsa_circ_0102049 is mapped to chromosome 14 on *ATL1* gene locus and has been originally found upregulated in OS [107,233]. On further studies, hsa_circ_0102049 correlated with poor prognosis, larger tumor size and pulmonary metastasis, and was found overexpressed on OS patient tissues (76 patients) and OS cell lines (U2OS, SaOS-2, MG-63, and HOS). Gain/loss of function experiments revealed accelerated cell proliferation, migration and invasion as well as attenuation of apoptosis upon hsa_circ_0102049 overexpression [233]. Additionally, this study provided evidence of hsa_circ_0102049 regulating MDM2 expression by sponging miR-1304-5p and revealed a novel hsa_circ_0102049/miR-1304-5p/MDM2 axis in OS.

On the other hand, a new study by Zhang et al. [234] found that silencing of hsa_circ_0102049 promotes proliferation, invasion, migration and cell cycle in OS cell line MG-63 by sponging miR-520g-3p and further modulating the miR-520g-3p/PLK2.Tap73 axis. Additional research is essential to elucidate hsa_circ_0102049 role on OS tumorigenesis.

### 3.4. Mechanism of Action and Potential Diagnostic and Prognostic Value of Important circRNAs in OS

A large number of circRNAs have been evaluated as potential biomarkers in OS tumorigenesis. However, only few of them hold promise in terms of prognostic and predictive value, as well as an effect on chemotherapy. In the first study, investigating the expression profile of circRNAs on chemoresistance to Doxorubicin (DXR) OS tissues (60 patient samples) and cell lines (MG-63, U2OS, KHOS and their (DXR) resistant pairs MG-63/DXR, U2OS/DXR and KHOS/DXR), Kun-Peng et al. detected 80 circRNAs dysregulated on chemoresistant cell lines and further studied the most upregulated circRNA, hsa_circ_0004674, which was related to poor prognosis [96]. Of the predicted targets of hsa_circ_0004674, miR-490-3p, miR-584-5p and miR-1254 are reported to have tumor suppressive roles including chemoresistance [235,236,237,238,239]. According to previous research findings of this group, chemoresistance in OS may be regulated by hsa_circ_0004674-miR-490-3p-ABCC2 and hsa_circ_0004674/miR-1254-EGFR pathways [96,240,241]. In addition, Bai et al. proposed a new mechanism of hsa_circ_0004674 promoting chemoresistance to DXR by regulating the miR-342-3p/FBN1 axis through the Wnt/β-catenin pathway [88].

On experiments aiming to elucidate the potential mechanisms of circRNAs and regulation of chemoresistance, using chemosensitive and chemoresistant paired cell lines (MG-63, U2OS, KHOS and (DXR) resistant pairs MG-63/DXR, U2OS/DXR and KHOS/DXR), hsa_circ_0081001 was detected significantly upregulated on OS tissues, cell lines and patient serum and correlated with poor survival [95]. Specifically, hsa_circ_0081001 expression was increased in higher stage patient groups, in chemoresistant patients and in lung metastasis patients. On multivariate analysis, chemoresistance, lung metastasis, Enneking stage and hsa_circ_0081001 overexpression were revealed as independent prognosticators [95]. Collectively, hsa_circ_0081001 overexpression showed a great prognostic value and may represent a biomarker in OS, superior to alkaline phosphatase and lactate dehydrogenase. Further studies by Wei et al. depicted that hsa_circ_0081001 knockdown enhanced methotrexate chemosensitivity in OS cell lines and chemoresistant pairs (U2OS-U2OS/R and HOS-HOS/R) and targeted miR-494-3p to elevate Translutaminase-2 (TGM2) levels [97].

The hsa_circ_0007534 was linked to poor prognosis in colorectal cancer [116]. Experiments on 57 OS patient tissues and OS cell lines HOS, SaOS-2, MG-63 and U2OS showed regulation of OS cell growth and apoptosis by hsa_circ_0007534 suggesting its possible prognostic value. hsa_circ_0007534 was upregulated in OS tissues and cell lines and facilitated OS tumorigenesis as well as tumor growth in xenograft mouse model. The oncogenic effects of hsa_circ_0007534 were attributed to its interaction with Phosphorylated AKT (pAKT) and Phosphorylated Glycogen Synthase Kinase-3β (pGSK-3β), as well as to the regulation of AKT/GSK-3β signaling pathway [115]. hsa_circ_0007534 high levels were correlated to tumor size and advanced histological grade, while hsa_circ_0007534 was determined as an independent prognosticator [115]. Another possible mechanism of action for hsa_circ_0007534 suggested by Zhang et al. is sponging of miR-219a-5p to upregulate Sex-Determining Region Y-box 5 (SOX-5) [242]. Recent evidence suggests that circPVT1 plays an important role in DXR and cisplatin chemoresistance. Kun-Peng conducted experiments in cell lines (MG-63, U2OS, KHOS and their (DXR) resistant pairs MG-63/DXR, U2OS/DXR and KHOS/DXR) that were also cross-resistant to cisplatin as well as tissues from 80 OS patients treated with regiments containing DXR and cisplatin [121]. circPVT1 was significantly upregulated in OS patient tissues, serum and cell lines and positively correlated to lung metastasis, advanced Enneking stage, shorter survival and chemoresistance. Furthermore, circPVT1 knockdown partly reversed DXR and cisplatin resistance in vitro and decreased ABCB1 expression which is highly expressed in drug-resistant cell lines, possibly by regulating the P-gp protein [243,244,245], further suggesting that circPVT1 inhibition mediates resensitization of OS cells to chemotherapy [121]. The effects of circPVT1 on DXR resistance were further validated in the study of Li et al. [98]. Detailed analysis indicated sponging of miR-137 by circPVT1 and subsequent regulation of TP53-regulated Inhibitor of Apoptosis 1 (TRIAP1), an inhibitor related to cisplatin sensitivity in human ovarian cancer [246].

Previous studies have shown that circ_ANKIB1 can act as an oncogene in OS [95]. miR-19b is documented to promote OS cell invasion migration and proliferation, and act as an oncogene in gliomas and colorectal carcinomas [247,248,249,250]. Mechanistic experiments conducted on OS cell lines MG-63, 143B, SaOS-2, U2OS and HOS showed that circ_ANKIB1 interacted with miR-19b and both of them were upregulated. Specifically, circ_ANKIB1 promotes miR-19b expression, as circ_ANKIB1 knockdown downregulated miR-19b expression and enhanced expression of SOCS3, respectively, with reduction in cell invasion. Collectively, it is suggested that circ_ANKIB1 and miR-19b promote OS cell invasion, while SOCS3 inhibits invasion, elucidating circ_ANKIB1 regulation on SOCS3/STAT3 pathway [125]. Another study by Zhu et al. confirmed the oncogenic role of circ_ANKIB1 in OS by regulating the miR-217/PAX3 axis [126].

Several studies have confirmed the ectopic expression of circ_001569 in OS. Zhang et al. reported circ_001569 upregulation, enhancement of cell proliferation and contribution to cisplatin resistance in OS patient tissues and cell lines (MG-63, U2OS). circ_001569 exerted its oncogenic role by activating Wnt/β-catenin signaling pathway, since increased circ_001569 upregulated phosphorylated GSK-3β and β-catenin and downregulated GSK-3β [89]. circ_001569 also correlated with distant metastasis and advanced tumor stage. Xiao et al. found that upregulated circ_001569 promotes proliferation, migration, invasion and EMT in OS by rescuing Flotillin-2 (FLOT2) expression via miR-185-5p sponging [169].

Conclusively, the abovementioned circRNAs regulate critical oncogenic pathways that justify their role as protooncogenes, and hsa_circ_0004674, hsa_circ_0081001, circPVT1 and circ_001569 were shown to exert important effect on OS prognosis, serving as potential biomarkers.

## 4. CircRNAs in Kaposi Sarcoma

Currently, the importance of circRNAs in immunology and significance in viral infections and host response has attracted the attention of investigators [251,252]. In 2018, Toptan et al. sequenced KSHV-infected cell lines BCBL-1 and BC-1 Primary Effusion Lymphoma (PEL) cell lines and detected constitutive expression of viral encoded circRNA circvIRF4, as well as numerous circRNAs from Polyadenylated Nuclear (PAN) RNA locus [253]. They confirmed the presence of circvIRF4 and circPAN/K7.3 isoforms in 4 out of 10 KS patient tissues, speculating that viral circRNAs participate in viral oncogenesis [253]. Tagawa et al. detected viral circRNAs in KSHV-infected primary Human Umbilical Vein Endothelial Cells (HUVECs) as well as in tissues from lymph nodes of patients suffering from KS, PEL or KSHV+ Castleman Disease [254]. This study also showed that infected cells stably expressing viral circRNAs differ in relative growth, compared to control cells in SLK cells-a KS cell line. They also identified a human circRNA, hsa_circ_0001400, induced by KSHV infection, with potential antiviral effect, and performed overrepresentation analysis that depicted enrichment of pathways involved in cancer or p53 signaling [254]. circvIRF4 and circPAN/K7.3 in KS tissues and serum were further investigated by the study of Abere et al., showing that 61/92 (66.3%) KS tissues were positive for KSHV circRNAs, namely 32/92 (34.8%) for circvIRF4, 49/92 (53.3%) for circPAN and 28/92 (30.4%) for circK7.3. A total of 5 out of 10 (50%) previously collected and stored KS patients’ sera were positive for viral circRNAs, while in fresh samples the respective percentage was 100% [255]. Moreover, Yao et al. provided an alternative mechanism of circRNA contribution to KS-driven oncogenesis, by identifying an upregulated cellular circular RNA, circARGEF1, which promotes cell invasion and angiogenesis in KS [256]. KS encodes the Viral Interferon Regulatory Factor 1 (vIRF1), which, apart from immune regulation possesses oncogenic properties, including p53 suppression and inhibition of TGF-β/Smad signaling [93,94]. Yao et al. demonstrated that vIRF1 and lymphoid enhancer binding factor 1 (Lef-1) binding induces circARGEF1 transcription, and circARGEF1 sponges miR-125a-3p to upregulate Glutaredoxin 3 (GLXR3) in vIRF1-transduced cells, KSHV-infected cells and KS tissues, promoting cell motility, proliferation and angiogenesis [256].

## 5. CircRNAs in Rhabdomyosarcoma

Rossi et al. utilized human primary wild-type myoblasts, ERMS RD cell line, ARMS RH4 cell line and RMS patient tissues to study the role of circZNF609 [257]. circZNF609 regulates cell cycle and immune response related genes in human primary myoblasts [76], and its silencing induces proliferation arrest. circZNF609 is upregulated in RMS tissues, RD and RRH4 cell lines, with particularly high levels in RH4 cell line. circZNF609 knockdown inhibited proliferation of RD but not of RH4 cells, and further analysis detected reduced p-Rb/Rb ratio and pAKT levels. After GO analysis, the downregulated genes were mainly associated with cell cycle progression, DNA replication and mitosis, while Gene Graph Enrichment Analysis (GGEA) detected genes involved in PI3K/AKT signaling pathway [257]. circZNF609 knockdown did not affect RH4 cells. A possible explanation is attributed to the upregulation of cell cycle-related targets of p53, that was found to be downregulated. Collectively, these results point to circZNF609 regulation of cell proliferation in RMS.

## 6. CircRNAs in Gastrointestinal Stromal Tumors

The study of Jia et al. used GIST-T1 and GIST-882 cell lines along with GIST patient tissues and conducted microarray analysis to detect 5770 circRNAs differentially expressed in GISTs. During expression profiling, they focused on three upregulated circRNAs (circ_0069765, circ_0084097 and circ_0079471) and their host genes (*KIT*, *PLAT* and *ETV1*) [258]. The importance of *KIT* mutation in GISTs is established as the main oncogenic event and involves ETS transcription factor ETV1. PLAT is enriched in blood vessel development involved in tissue specificity in GISTs and interacts with VEGFC, PGF and CHD7 [259]. The researchers also identified miRNAs targeted by *KIT*, *PLAT* and *ETV1* and by circRNAs circ_0069765, circ_0084097 and circ_0079471, and constructed specific regulatory networks that may have important regulating roles in GISTs [258].

## 7. Conclusions–Perspective

CircRNAs have currently emerged as a promising research field in oncology, due to their outstanding properties and their crosstalk with multiple regulatory networks, signaling pathways, cellular processes and developmental events. Apart from their direct effect in tumorigenesis, circRNAs participate in immune modulation, which interferes with tissue microenvironment, being critical for tumor development. CircRNAs exhibit distinct expression pattern, stability and abundant detection levels in body fluids, such as blood, plasma or saliva, as well as in exosomes circulating in blood [260,261], making their function as potential biomarkers or as molecular markers to support diagnosis very attractive. The oncogenic implication of circRNAs as tumor promoters or tumor suppressors renders them a possible therapeutic target, either by the use of siRNAs complementary to the BSJ or by Antisense Oligonucleotides (ASOs) binding to the respective pre-mRNA and inhibition of oncogenic circRNAs, or by inducing expression of tumor suppressor circRNAs [262]. Modulation of circRNA levels may also be sensitive as a surrogate method to increase chemosensitivity in some tumors. Moreover, numerous studies have indicated the prognostic value of circRNAs. Future studies are highly demanded to improve detection methods as well as develop further clinical applications of these pivotal players of carcinogenesis.

To our knowledge, this is the first comprehensive literature review of circRNAs implication in all sarcoma types. Research in sarcomagenesis has been mainly focused on OS, indicating many circRNAs as possible therapeutic targets as well as prognostic biomarkers. Moreover, detection of certain circRNAs, such as hsa_circ_0081001 and circPVT1 in serum can be used for post-operative patient screening, aiding in the early detection of relapse. However, a small number of circRNAs in OS has provided ambiguous results regarding their functional role and will need further investigation. Of note, it is evident that different circRNAs play critical roles in each sarcoma type. Specifically, circRNAs present in KS are mainly of viral origin while circZNF609 detected in RMS and circ_0069765, circ_0084097 and circ_0079471 detected in GISTs have not been identified in OS. Conclusively, current data shows that circRNAs are involved in numerous significant oncogenic mechanisms that are implicated in various sarcoma types. Further research is highly demanded to investigate the pathogenic mechanisms in which circRNAs are involved in each sarcoma subtype.

## Figures and Tables

**Table 1:** Upregulated circRNAs in OS.

CircRNA	Sample Types	Targeted Molecules/Axis	Involved Signaling Pathway	Prognostic Implications	Role in OS Tumorigenesis	Other Tumor Types Tested
circMMP9 [99]	MG-63, SaOS-2, HOS, U2OS, hFOB1.19, patient tissues	miR1265/CHI3L1 ^1^	-	Advanced tumor stage, adverse prognosis	Cell proliferation, invasion, migration, apoptosis inhibition	Glioblastoma [100]
hsa-circ-0016347 [101,102]	MG-63, SaOS-2, hFOB1.19, patient tissues, mouse xenograft model	miR-214/Caspase-1	-	-	Cell proliferation, invasion, metastasis	-
	OS cell lines, mouse xenograft model	miR-1225-3p/KCNH1 ^2^			Tumor progression	
cir-GLI2 [103]	MG-63, SaOS-2, HOS, U2OS, hFOB1.19, HEK293, patient tissues	miR-125b-5p	-	-	Cell proliferation, invasion, migration	-
hsa_circ_0009910 [104]	MG-63, SaOS-2, U2OS, hFOB1.19, patient tissues	miR-449a/IL6R ^3^	JAK1/STAT3	-	Cell proliferation, cell cycle arrest, apoptosis inhibition	-
hsa_circ_0001564 [105]	MG-63, SaOS-2, HOS, U2OS, patient tissues	miR-29c-3p	-	-	Cell proliferation, cell cycle arrest, apoptosis inhibition	-
hsa_circ_0004674 [88,96]	KHOS, KHOS/DXR, U2OS, U2OS/DXR, MG-63, MG-63/DXR, patient tissues	miR-490-3p-ABCC2, miR-1254-EGFR	-	Adverse prognosis	Doxorubicin chemoresistance	-
	KHOS, KHOS/DXR, U2OS, U2OS/DXR, patient tissues, mouse xenograft model	miR-342-3p/FBN1	Wnt/β-catenin		-	
circRNA_100876 [106]	MG-63, OS-732, SaOS-2, HOS, U2OS, 143B, patient tissues, mouse xenograft model	miR-136	-	Large tumor size, poor tumor differentiation degree	Cell proliferation, cell cycle progression, apoptosis inhibition	Non-small cell lung cancer [107]
hsa_circ_0081001 [95,97]	MG-63, MG-63/DXR KHOS, KHOS/DXR, U2OS, U2OS/DXR, patient tissues and serum	-	-	Advanced tumor stage, lung metastasis, advanced Enneking stage, independent prognosticator	Tumor growth, methotrexate chemoresistance	-
	HOS, HOS/MTXR, U2OS, U2OS/MTXR, patient tissues, mouse xenograft model	miR-494-3p/TGM2 ^4^		-	-	
circFAT1 [108,109]	SJSA-1, MG-63, SaOS-2, HOS, U2OS, 143B, hFOB1.19, patient tissues, mouse xenograft model	miR-375/YAP1	Hippo	-	Cell proliferation, migration, apoptosis inhibition	Gastric cancer [110]
	SW1353, MG-63, hFOB1.19	miR-181b/HK2 ^5^	-		Cell growth, migration, metastasis	
circ_2137, circ_20403 [111,112]	Patient tissues	-	Phosphatidyl-inositol signaling pathway, Inositol phosphate metabolism	-	-	-
	MG-63, U2OS, 143B, G292, hFOB1.19, patient tissues, mouse xenograft model	circ_2137/miR-433-3p/IGF1R	-		Invasion, cell cycle progression, apoptosis inhibition	
circ_0001721 [113,114]	MG-63, SaOS-2, HOS, U2OS, hFOB1.19, patient tissues, mouse xenograft model	miR-569, miR-599	-	Large tumor size, high differentiation grade, adverse 5-year prognosis	Cell proliferation	-
hsa_circ_0007534 [115,116]	MG-63, SaOS-2, HOS, U2OS, hFOB1.19, patient tissues, mouse xenograft model	-	AKT/GSK-3β	Large tumor size, poor differentiation grade, independent prognosticator	Tumor growth, apoptosis inhibition	Pancreatic ductal carcinoma [117], glioma [118], colorectal cancer [116], breast cancer [119]
	MG-63, HOS, U2OS, 143B, hFOB1.19, patient tissues, mouse xenograft model	miR-219a-5p/SOX-5 ^6^	-	-	Cell proliferation, colony formation, migration, invasion	
circNASP [120]	MG-63, 143B, patient tissues	miR-1253/FOXF1	-	Large tumor size, lung metastasis	Cell proliferation, cell cycle progression, invasion	-
circPVT1 [98,121]	MG-63, SaOS-2, KHOS, U2OS, patient tissues and serum	P-gp/ABCB1	-	Lung metastasis, advanced Enneking stage, poor survival	Tumor growth, doxorubicin chemoresistance	Gastric cancer [122]
	KHOS, U2OS, 293T, patient tissues, mouse xenograft model	miR-137/TRIAP1 ^7^		-	-	
hsa_circ_0000885 [123]	patient tissues and serum	-	-	Advanced Enneking stage, lung metastasis	-	-
circ-0001785 [124]	MG-63, SJSA-1, SaOS-2, HOS, U2OS, hFOB1.19, mouse xenograft model	miR-1200/HOXB2	Bcl-2 family, PI3K/AKT/mTOR	-	Cell proliferation, apoptosis inhibition	-
circ_ANKIB1 [95,125,126]	MG-63, SaOS-2, HOS, U2OS, 143B, hFOB1.19	miR-19b/SOCS3	SOCS3/STAT3	-	Cell proliferation, invasion	-
	HOS, U2OS, hFOB1.19, patient tissues, mouse xenograft model	miR-217/PAX3	-		Cell proliferation, migration, invasion, tumor growth, apoptosis inhibition	
circMYO10 [127]	SJSA-1, MG-63, HOS, U2OS, 143B, hFOB1.19, patient tissues, mouse xenograft model	miR-370-3p/RUVBL1	β-catenin/LEF1/c-MYC	-	Cell proliferation, EMT	-
circTADA2A [128]	HEK-293, SJSA-1, MG-63, HOS, U2OS, 143B, patient tissues, mouse xenograft model	miR-203a-3p/CREB3	-	-	Cell proliferation, migration, invasion	-
circ-0003998 [91]	MG-63, SaOS-2, U2OS, 143B, hFOB1.19, patient tissues	miR-197-3p/KLF10 ^8^	TGF-β	Poor overall survival	Cell proliferation, invasion	Non-small cell lung cancer [129]
circ_0001658 [130]	MG-63, SaOS-2, HOS, U2OS, 143B, hFOB1.19, patient tissues	miR-382-5p/YB-1	-	Early relapse	Cell proliferation, migration, metastasis, apoptosis inhibition	-
circ_ORC2 [131]	MG-63, SaOS-2, HOS, U2OS, 143B, hFOB1.19	miR-19a/PTEN ^9^	PI3K/AKT	-	Cell proliferation, invasion, apoptosis inhibition	-
circSAMD4A [132]	MG-63, HOS, U2OS, 143B, hFOB1.19, patient tissues, mouse xenograft model	miR-1244/MDM2	-	Poor overall survival	Cell proliferation, metastasis	-
has-circ-0001146 [133]	MG-63, 143B, hFOB1.19, patient tissues, mouse xenograft model	miR-26a-5p/MNAT1	-	-	Cell proliferation, viability, invasion, apoptosis inhibition	-
circITGA7 [134]	SW1353, MG-63, HOS, U2OS, patient tissues	miR-370/PIM1	-	-	Cell proliferation, migration, metastasis	Colorectal cancer [135], thyroid cancer [136]
hsa_circ_0003732 [137]	MG-63, SaOS-2, HOS, U2OS, hFOB1.19, patient tissues	miR-545/CCNA2	-	Poor prognosis	Cell proliferation	-
circEIF4G [138]	MG-63, HOS, patient tissues	miR-218	EGFR, PI3K/AKT, ErbB	-	Cell proliferation, migration, invasion	Cervical cancer [139]
circSMARCA5 [140]	MG-63, HOS	-	-	-	Cell proliferation, cell cycle progression, adhesion, migration, metastasis	Prostate cancer [141], hepatocellular carcinoma [142], cervical cancer [143], glioma [144], intrahepatic cholangiocarcinoma [145]
circ_001621 [146]	MG-63, U2OS, hFOB1.19, patient tissues, mouse xenograft model	miR-578/VEGF ^10^, CDK4, MMP9	-	Advanced tumor stage, poor survival, metastasis	Cell proliferation, migration	-
hsa_circ_0000282 [147]	SOSP-9607, MG-63, U2OS, 143B, hFOB1.19, patient tissues	miR-192/XIAP ^11^	-	High tumor differentiation grade, advanced Enneking stage	Cell proliferation, apoptosis inhibition	-
circEPSTI1 [148]	MG-63, U2OS, mouse xenograft model	miR-892b/MCL1	-	-	Cell proliferation, migration, invasion	Breast cancer [149]
hsa_circ_0000073 [150]	MG-63, MG-63/DXR, HOS, U2OS, U2OS/DXR, 143B, patient tissues	miR-145-5p/NRAS, miR-151-3p/NRAS	-	-	Cell proliferation, migration, invasion, methotrexate chemoresistance	-
hsa_circ_0136666 [151]	OS cell lines, hFOB1.19, patient tissues, mouse xenograft model	miR-593-3p/ZEB2	-	Large tumor size, adnanved tumor stage, poor survival	Cell proliferation, migration, invasion, apoptosis inhibition	Colorectal cancer [152], breast cancer [153]
circ_100284 [154]	MG-63, SaOS-2, U2OS, hFOB1.19, patient tissues	LSD1 ^12^-EZH2 ^13^/PTEN	-	Large tumor size, lung metastasis, poor survival	Cell viability, invasion, cell cycle progression, apoptosis inhibition	-
circ-0060428 [155]	SaOS-2, HOS, U2OS, 143B, hFOB1.19	miR-375/RPBJ/Bax-bcl-2-cleaved-caspace-3	-	-	Cell proliferation, apoptosis inhibition	-
circ_0010220 [156,157]	MG-63, SaOS-2, HOS, U2OS, 143B, hFOB1.19	miR-503-5p/CDCA4 ^14^	-	-	Tumor growth, migration, invasion	-
	HOS, U2OS, hFOB1.19 patient tissues, mouse xenograft model	miR-198/Syntaxin_6		Poor survival	Cell proliferation, migration, invasion,	
circ-XPO1 [158]	MG-63, SaOS-2, HOS, U2OS, hFOB1.19, patient tissues	miR-23a-3p/XPO1, miR-23b-3p/XPO1, miR-130a-5p/XPO1, miR-23c/XPO1	-	Poor survival	Tumor growth, invasion, apoptosis inhibition	-
hsa_circ_0032462, hsa_circ_0028173, hsa_circ_0005909 [159]	MG-63, HOS, U2OS, 143B, hFOB1.19	has-miR-338-3/CADM1 ^15^, has-miR-142-5p/CADM1	Cell cycle pathway, cell adhesion molecules pathway, p53 signaling, oxidative phosphorylation pathway, cytokine-cytokine receptor interaction pathway	-	-	-
CDR1as [160]	MG-63, SaOS-2, HOS, U2OS, 143B, hFOB1.19, patient tissues, mouse xenograft model	miR-7/*EGFRA*, miR-7/*CCNE1*, miR-7/*PI3KCD*, miR-7/*RAF1*, N-cadherin, E-cadherin	-	Large tumor size, advanced Enneking stage, distant metastasis	EMT, cell migration, tumor growth	Hepatocelular carcinoma [161,162], glioblastoma [163], breast cancer [164], ovarian cancer [165], urothelial cancer [166], gastric cancer [167], non-small cell lung cancer [168]
circ_001569 [89,169]	MG-63, U2OS, hFOB1.19, patient tissues	GSK-3β, β-catenin	Wnt/β-catenin	Advanced tumor stage, distant metastasis	Cell proliferation, cisplatin chemoresistance	Colorectal cancer [170]
	MG-63, SaOS-2, HOS, hFOB1.19, patient tissues, mouse xenograft model	miR-185-5p/FLOT2 ^16^	-	-	Cell proliferation, migration, invasion, EMT	
circ_0000502 [171]	MG-63, SaOS-2, HOS, U2OS, patient tissues, mouse xenograft model	miR-1238	-	-	Cell proliferation, migration, invasion, apoptosis inhibition	-
circLRP6 [172]	MG-63, HOS, U2OS, SaOS-2, patient tissues	LSD1, EZH2	-	Shorter disease-free survival and overall survival	Cell proliferation, migration, invasion	-
hsa_circ_0000285 [92]	MG-63, SaOS-2, HOS, U2OS, hFOB1.19, mouse xenograft model	hsa-miR-599/TGF-β2	-	-	Cell proliferation and migration	-
circ-XPR1 [173]	MG-63, U2OS, patient tissues	miR-214-5p/DDX5	-	Poor overall survival and disease-free survival	Cell proliferation	-
circUBAP2 [85,174]	MG-63, U2OS, hFOB1.19, patient tissues, mouse xenograft model	miR-143/bcl-2	-	Advanced tumor stage, poor survival, poor prognosis	Tumor growth, apoptosis inhibition	Renal cancer [175] esophageal squamous carcinoma [176], triple-negative breast cancer [177], ovarian cancer [178]
	MG-63, HOS, SaOS-2, U2OS, patient tissues	miR-204-3p/HMGA2	-	Poor survival	Cell proliferation, migration, invasion, apoptosis inhibition	
circ_ARF3 [179]	MG-63, SaOS-2, U2OS, patient tissues, mouse xenograft model	miR-1299/CDK6	-	-	Cell growth, cell cycle progression	-
circ-NT5C2	SOSP-9607, MG-63, SaOS-2, U2OS, patient tissues, mouse xenograft model	miR-448 [180]	-	Advanced Enneking stage, lung metastasis	Cell proliferation, invasion	-
circRNA-0008717 [113]	SW1353, MG-63, SaOS-2, HOS, U2OS, hFOB1.19, patient tissues	miR-203/Bmi-1	-	Poor overall survival and progression-free survival	Cell proliferation, invasion	-
hsa_circRNA_103801 [181,182]	MG-63, HOS, U2OS, U2OS/MTX300, ZOS, ZOS-M, 143B, hFOB1.19, patient tissues	-	HIF-1, VEGF, angiogenesis pathway, Rap1 signaling pathway, PI3K/AKT signaling pathway	-	-	-
	OS cell lines, patient tissues	miR-338-3p/HIF1-Rap1	PI3K/AKT	-	Cell proliferation, migration, invasion	
hsa_circ_0032463 [183,184]	SOSP-9607, HOS, U2OS, SaOS-2, patient tissues	miR-330-3p/PNN ^17^	-	-	Cell proliferation, viability, invasion, adhesion, apoptosis inhibition	-
	SOSP-9607, SW1353, SaOS-2, HOS, U2OS, hFOB1.19, patient tissues	miR-498/LEF1	-	-	Tumor proliferation, migration, apoptosis inhibition	
circCAMSAP1 [185]	HOS, U2OS, 143B, hFOB1.19, patient tissues, mouse xenograft model	miR-145-5p/FLI1 ^18^	-	-	Cell growth, apoptosis inhibition, migration, invasion	-
circNRIP1 [56]	MG-63, U2OS, patient tissues	METTL3/circNRIP1/miR-199a/FOXC2	-	-	Cell proliferation, migration, apoptosis	Gastric cancer [186], renal cancer [187], cervical cancer [188]
circSIPA1L1 [189]	MG-63, SJSA-1, HOS, U2OS, 143B, hFOB1.19, patient tissues, mouse xenograft model	miR-411-5p/RAB9A	-	-	Cell vitality, invasion, migration, proliferation	-
circPRDM2 [190]	MG-63, MG-63/DXR, KHOS, KHOS/DXR, hFOB1.19, patient tissues, mouse xenograft model	miR-760/EZH2	-	-	Cell migration, invasion, colony formation, doxorubicin chemoresistance, apoptosis inhibition	-
circRAB3IP [191]	MG-63, HOS, U2OS, 143B, patient tissues, mouse xenograft model	miR-580-3p/TWIST ^19^	-	Advanced tumor stage, distant metastasis	Cell proliferation, migration, invasion	-
circ_CDK14 [192]	OS cell lines, patient tissues	miR-520a-3p/GAB1 ^20^	-	-	Cell proliferation, metastasis, tumorigenesis, apoptosis	-
circ-CHI3L1.2 [193]	OS cell lines	miR-340-5p/LPAATβ ^21^	-	-	Cell migration, invasion, EMT, cisplatin chemoresistance	-
circ_0000337 [194]	MG-63, HOS, U2OS, 143B, hFOB1.19	miR-4458/BACH1	-	-	Cell growth, migration	Esophageal squamous carcinoma [195]
circ_0000527 [196]	MG-63, SaOS-2, HOS, U2OS, hFOB1.19, patient tissues	miR-646/ARL2	-	-	Cell growth, cell cycle, inflammation	Retinoblastoma [197]
circ_001422 [198]	MNNG, MG-63, SaOS-2, U2OS, 143B, hFOB1.19, patient tissues, mouse xenograft model	miR-195-5p/FGF2	PI3K/AKT	Advanced tumor stage, large tumor size, distant metastasis, poor overall survival	Cell proliferation, metastasis, apoptosis inhibition	-
hsa_circ_0051079 [199]	MG-63, SaOS-2, HOS, KHOS, U2OS, 143B, patient tissues, mouse xenograft model	miR-26a-5p/TGF-β1	-	-	Tumor proliferation, metastasis	-
circ_0056285 [200]	MG-63, HOS, U2OS, 143B, hFOB1.19, patient tissues and serum, mouse xenograft model	miR-1244/TRIM44 ^22^	-	-	Cell proliferation, glycolysis, apoptosis inhibition	-
circ_0084582 [201]	MG-63, U2OS, hFOB1.19, patient tissues, mouse xenograft model	miR-485-3p/JAG1 ^23^	Notch pathway	-	Cell proliferation, cell cycle progression, migration, invasion, angiogenesis	-
circPOK [202]	-	ILF2/3 complex	-	-	Tumorigenic	-

^1^ Chitinase-3-like Protein 1; ^2^ Potassium Voltage-Gated Channel Subfamily H Member 1; ^3^ Interleukin 6 Receptor; ^4^ Translutaminase-2; ^5^ Human Glandular Kallilrein2; ^6^ Sex-Determining Region Y-box 5; ^7^ TP53-regulated Inhibitor of Apoptosis 1; ^8^ Kruppel-like Factor 10; ^9^ Phosphate and Tensin Homolog; ^10^ Vascular Endothelial Growth Factor; ^11^ X-linked Inhibitor of Apoptosis Protein; ^12^ Lysine-Specific Histone Demethylase 1A; ^13^ Enhancer of Zeste Homolog 2; ^14^ Cycle-Associated Protein 4; ^15^ Cell Adhesion Molecule 1; ^16^ Flotillin-2, ^17^ Pinin Desmosome Associated Protein; ^18^ Friend Leukemia Virus Integration 1, ^19^ Twist Family BHLH Transcription Factor; ^20^ GRB2 Associated Binding Protein 1; ^21^ Lysophosphatidic Acid Acyltransferase β; ^22^ Tripartite Motif Containing 44; ^23^ Jagged1.

**Table 2:** Downregulated circRNAs in OS.

CircRNA	Sample Types	Targeted Molecules/Axis	Involved Signaling Pathway	Prognostic Relevance	Role in OS Tumorigenesis	Other Tumor Types Tested
circ-LARP4 [203]	MG-63, SaOS-2, patient tissues	miR-424	-	Low Enneking stage, tumor cell necrosis after adjuvant chemotherapy, prolonged disease-free survival and overall survival	Increasing chemosensitivity to cisplatin and doxorubicin	Gastric cancer [204], hepatocellular carcinoma [205], ovarian cancer [206]
hsa_circ0021347 [207]	MG-63, patient tissues	B7-H3	-	Low tumor stage, low Enneking stage, prolonged survival	-	-
hsa_circ_0001258 [208]	MG-63, MG-63/DXR KHOS, KHOS/DXR, U2OS, U2OS/DXR, patient tissues	miR-744-3p/GSTM2	-	-	Cell viability, increasing chemosensitivity to doxorubicin	-
has_circ_0000190 [209]	MG-63, SaOS-2, KHOS, SJSA1, patient tissues, mouse xenograft model	miR-76-5p/TET1	-	-	Cell proliferation, migration, and invasion inhibition	Gastric cancer [210], plasma cell myeloma [211]
circ_0046264 [212]	MG-63, HOS, U2OS, 143B, patient tissues	miR-940/SFRP1 ^1^	-	Low tumor size and Ki67 proliferation index	Cell proliferation, migration, and invasion inhibition	Lung cancer [213]
circ_0001105 [214]	MG-63, U2OS, 143B, patient tissues, mouse xenograft model	miR-766/YTHDF2	-	Prolonged survival	Cell viability, migration, and invasion inhibition	-
circ_32279, circ_24831 [111]	Patient tissues	-	Phosphatidylinositol signaling pathway, inositol phosphate metabolism	-	-	-
hsa_circRNA_104980 [181]	MG-63, HOS, U2OS, U2OS/MTX300, ZOS, ZOS-M, 143B, hFOB1.19, patient tissues	hsa-miR-1298-3p, hsa-miR-660-3p	Phosphoric ester hydrolase activity, carbohydrate derivative binding	-	-	-
hsa_circ_0000658 [215]	MG-63, SJSA-1, SaOS-2, HOS, U2OS, hFOB1.19, patient tissues, mouse xenograft model	miR-1227/IRF2 ^2^	-	-	Cell cycle, proliferation, invasion, and migration inhibition	-
circMTO1 [216]	MG-63, SaOS-2, HOS, U2OS, hFOB1.19, patient tissues	miR-630/KLF6	-	Low Enneking stage, prolonged overall survival	Proliferation, migration and invasion inhibition, apoptosis induction	Breast cancer [217], cervical cancer [218], hepatocellular carcinoma [219]
circVRK1 [220]	MG-63, SaOS-2, HOS, U2OS, 143B, hFOB1.19, patient tissues, mouse xenograft model	miR-337/ZNF652 ^3^	-	Low levels correlate to poor prognosis and distant metastasis	Growth, migration, invasion inhibition	Breast cancer [221], esophageal cancer [222]
circ_WWC3 [223]	MG-63, SaOS-2, HOS, U2OS, hFOB1.19, patient tissues, mouse xenograft model	miR-421/PDE7B ^4^	-	-	Cell growth, migration, invasion inhibition, apoptosis induction	-

^1^ Secreted Frizzled Related Protein 1; ^2^ Interferon Regulatory Factor 2; ^3^ Zinc-Finger Protein 652; ^4^ Phosphodiesterase 7B.

## Abbreviations

ADAR1	Adenosine Deaminase RNA-Specific Binding Protein
Ago2	Argonaute 2
APC	Adenomatous Polyposis Coli Protein
ARL2	ADP-Ribosylation Factor-like Protein 2
ARMS	Alveolar RMS
ASOs	Antisense Oligonucleotides
ATP	Adenosine Triphosphate
BACH1	BTB Domain and CNC Homolog 1
BSJ	Back-Splicing Junction
CADM1	Cell Adhesion Molecule 1
CDCA4	Cycle-Associated Protein 4
CDR1as	Cerebellar Degeneration-Related Protein 1 Transcript
CHI3L1	Chitinase-3-like Protein 1
circHIPK2	CircRNA-Homeodomain-Interacting Protein Kinase-2
circRNAs	Circular RNAs
ciRNAs	Intronic circRNAs
dsRBPs	Double-Strand RNA-Binding Proteins
DXR	Doxorubicin
EcircRNAs	Exonic circRNAs
EIcircRNAs	Exon-Intron circRNAs
EMT	Epithelial-Mesenchymal Transition
ERMS	Embryonal RMS
EZH2	Enhancer of Zeste Homolog 2
f-circRNAs	Fusion circRNAs
FISH	Fluorescence In Situ Hybridization
FLI1	Friend Leukemia Virus Integration 1
FLOT2	Flotillin-2
FUS/TLS	Fused in Sarcoma/Translocated in Liposarcoma
G3BP1	Ras Gtpase-Activating Protein-Binding Protein 1
GAB1	GRB2 Associated Binding Protein 1
GGEA	Gene Graph Enrichment Analysis
GISTs	Gastrointestinal Stromal Tumors
GLXR3	Glutaredoxin 3
HK2	Human Glandular Kallilrein2
HRSP12	Heat-Responsive Protein 12
HUVECs	Human Umbilical Vein Endothelial Cells
IL6R	Interleukin 6 Receptor
IRES	Internal Ribosome Entry Site
IRF2	Interferon Regulatory Factor 2
JAG1	Jagged1
KCNH1	Potassium Voltage-Gated Channel Subfamily H Member 1
KLF10	Kruppel-like Factor 10
KS	Kaposi Sarcoma
KSHV/HHV-8	Kaposi’s Sarcoma-Associated Herpesvirus
LPAATβ	Lysophosphatidic Acid Acyltransferase β
LSD1	Lysine-Specific Histone Demethylase 1A
m^6^A	N6-Methyladenosine
MBNL1	Muscleblind-like Protein 1
miRNA	microRNA
mRNA	Messenger RNA
ncRNAs	Non-coding RNAs
nt	Nucleotide
ORF	Open Reading Frame
OS	Osteosarcoma
pAKT	Phosphorylated AKT
PAN	Polyadenylated Nuclear
PDE7B	Phosphodiesterase 7B
PEL	Primary Effusion Lymphoma
PFS	Progression-Free Survival
PNN	Pinin Desmosome Associated Protein
Pol II-U1	RNA Polymerase II-U1
PRMS	Pleomorphic RMS
PTEN	Phosphate and Tensin Homolog
RBPs	RNA-Binding Proteins
RMS	Rhabdomyosarcoma
RNase	Ribonuclease
SDH	Succinate Dehydrogenase
SFRP1	Secreted Frizzled Related Protein 1
siRNAs	Small Interfering RNAs
snRNPs	Small Nuclear Ribonucleoproteins
SOX-5	Sex-Determining Region Y-box 5
STAT3	Signal Transducer and Activator of Transcription 3
TGF-β	Transforming Growth Factor Beta
TGM2	Translutaminase-2
TRIAP1	TP53-regulated Inhibitor of Apoptosis 1
tricRNAs	tRNA Intronic circRNAs
TRIM44	Tripartite Motif Containing 44
TWIST	Twist Family BHLH Transcription Factor
UPF1	ATP-Dependent RNA Helicase Upstream Frameshift 1
UPS	Undifferentiated Pleomorphic Sarcoma
VEGF	Vascular Endothelial Growth Factor
vIRF1	Viral Interferon Regulatory Factor 1
XIAP	X-linked Inhibitor of Apoptosis Protein
YTHDF2	YTH N6-Methyladenosine RNA Binding Protein 2
ZNF652	Zinc-Finger Protein 652
